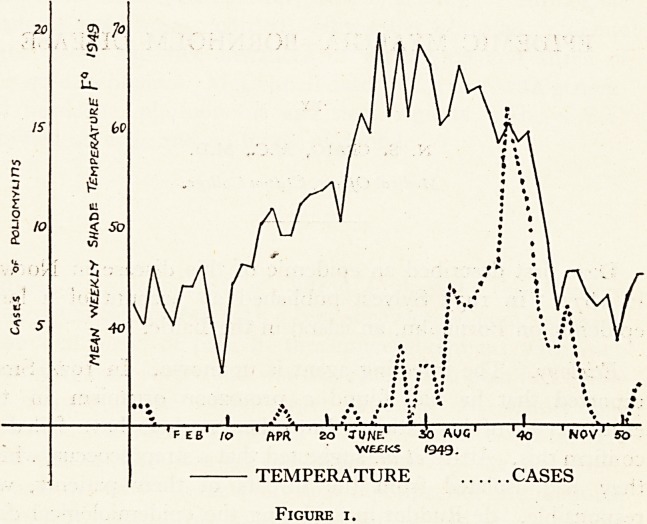# Poliomyelitis in Bristol, 1949

**Published:** 1950-04

**Authors:** James Macrae

**Affiliations:** Resident Physician, Ham Green Hospital, Bristol


### The Bristol Medico-Chirurgical Journal

***A Journal of the Medical Sciences for the***

### West of England and South Wales

*" Scire est nescire, nisi id me*
*Scire alius sciret."*

APRIL, 1950

### POLIOMYELITIS IN BRISTOL, 1949

BY

JAMES MACRAE, M.D., F.R.F.P.S., D.P.H.
*Resident Physician, Ham Green Hospital, Bristol*

In 1947 I gave an account of the manner in which the poliomyelitis epidemic of that year, the first really serious epidemic seen in Great Britain, had affected Bristol.* The 1947 incidence of the disease was very light. But we were hardly hit in 1949. Since Ham Green Hospital admits the majority of cases occurring in the Bristol area our admission† figures should be a measure of the local incidence.

TABLE I
POLIOMYELITIS INCIDENCE

| | 1947 | | 1948 | | 1949 | |
|---|---|---|---|---|---|---|
| Britain—Cases (*Lancet*, 1950) | 7,766 | *18* | 1,859 | *4* | 5,800 | *13* |
| Ham Green Hospital—Cases | 23 | *4* | 29 | *6* | 125 | *25* |

Rate per 100,000 in italics

* *Jl.*, 1947, LXIV, p. 101.
† Other admissions, excluding transfers: Bristol Royal Infirmary, 12; Children's Hospital, 37; Southmead Hospital, 14.
City and County of Bristol: 130 cases, 85 males; 44 per cent. under 5 years, 33 per cent. 5–14 years. 5 deaths.

Casey (1946) and Hare (1949) believe that for every case with paralysis there are ninety-nine abortive infections. If we assume this is true also in Britain, and since the majority of our cases were paralytic, the numbers do not indicate the actual local prevalence of the infection. Indeed, the cases we are considering now are the unlucky people who developed the rather unusual complication of paralysis!

*Cases admitted to Ham Green Hospital.* Of 183 patients sent in as poliomyelitis the diagnosis was confirmed in 107. Eighteen other patients were found to be suffering from the disease although admitted under some other diagnosis. Among the 125 confirmed cases there were eight deaths, and eighteen abortive cases.

TABLE II

AGE AND SEVERITY

Males, 74 (6). Females, 51 (2)

| Age | 0–1 | 1–4 | 5–14 | 15–24 | 25 + | Total |
|---|---|---|---|---|---|---|
| Abortive .. | — | 1 | 7 | 6 | 4 | 18 |
| Slight .. | — | 6 | 4 | 2 | 4 | 16 |
| Moderate .. | 2 | 21 | 19 | 8 | 5 | 55 |
| Severe .. | 2 (1) | 9 | 10 (1) | 8 (4) | 7 (2) | 36 (8) |
| | 4 (1) | 37 | 40 (1) | 24 (4) | 20*(2) | 125 |

Deaths in parentheses. * Four over 35.

*Diagnosis.* There is little to add to the lengthy literature on diagnosis, and indeed seldom any difficulty is encountered in making a diagnosis in the presence of paralysis. A febrile illness with some meningeal signs followed by paralysis is the usual story of poliomyelitis. Occasionally the same picture can be noted as a result of Landry's paralysis or mumps encephalo-meningitis, and two cases of each of these conditions appeared among our cases originally thought to be poliomyelitis. Continued observation, careful history-taking and repeated examination of the cerebro-spinal fluid will usually show the true diagnosis.

While the diagnosis of the paralysed case might be easy, the same cannot be said for the non-paralytic variety. This diagnosis must always be a clinical presumption. Often the patient appears as suffering from meningitis; examination of the cerebro-spinal fluid suggests an encephalitis and sometimes the clinician is doubtful if the case is not tuberculous meningitis at an early stage. The considerable number of cases in this series sent to hospital with the wrong diagnosis is due to this difficulty in diagnosis of the non-paralytic disease. Nobody can say that such a case is not going to develop paralysis: indeed one of our cases remained quite ill in the preparalytic state for seventeen days and thereafter was severely paralysed. On the other hand, there are many conditions which can simulate non-paralytic poliomyelitis. Hence, in the diagnosis of this variety of the disease we regarded pleocytosis and increased protein in the cerebro-spinal fluid as necessary pointers towards a diagnosis. It might be said that abortive poliomyelitis may not evoke a cerebro-spinal fluid response; this is quite possibly true, since three of our *paralysed* cases showed no such response throughout the illness. However, in deciding on such an evasive diagnosis and in absence of any method of demonstrating the virus, one must start on some sort of firm ground. Then, having eliminated the presence of increasing blood antibody titres against chorio-meningitis, leptospirosis, mumps, mononucleosis, ornithosis-lymphogranuloma and Q fever, we observed the case and finally, in default, called it abortive infantile paralysis. The lack of a positive method of diagnosis of these cases is unfortunate.

The differential diagnosis of poliomyelitis, as evidenced by the true nature of the disease in the seventy-six patients admitted with this diagnosis, covers a wide field and is further evidence of the concern that the nonparalytic case causes. Tonsillitis, with twenty-three cases, heads the list, other central nervous diseases eighteen cases, septicaemic conditions six cases, and for the rest, odd cases of osteomyelitis, arthritis, rheumatism, fibrositis, otitis media, pneumonia, migraine, hysteria, sunburn, puerperal sepsis, pyelitis, nephritis, constipation and diabetic coma.

The course of the illness is largely unpredictable; the duration and severity of the preparalytic stage varies within wide limits and these variations do not bear any recognizable relation to the extent of any subsequent paralysis. When paretic signs developed, most commonly they were maximal at the onset, but seventeen cases continued to have spreading paralyses over a period of days; one indeed continued so for eight days. Generally speaking, paralysis ceased spreading when the temperature fell to normal. Thereafter each case went its own way; some with extensive initial paralysis cleared up rapidly; most showed an improvement during the first post-paralytic week and all had some improvement within a month. As with the onset, the clearance of paralysis did not bear distinct relation to the severity of the illness or the extent of the initial paralysis. The distribution of the paralysis seemed to be an accidental phenomenon in this series and, although close interest was displayed, any connection with exercise was not well displayed. Indeed sixteen of the severely paralysed cases had led particularly restrained lives previous to infection. One of these was an advanced case of pulmonary tuberculosis in our own sanatorium. Two other local patients, previously long immobilized in plaster beds, developed severe paralysis (personal communication).

There were five instances of two cases occurring in the same household and one instance of three simultaneous cases, all with paralysis.

*The Deaths.* The death rate of our cases is relatively low. All those who died had severe paralysis and four of them died of steadily progressive paresis. Two patients died exceedingly suddenly of cardiac failure ; they had had no respiratory paresis although other paralysis was widespread. One patient died as a result of pharyngeal paralysis; he was in the artificial respirator and all efforts to keep his air passages dry were of no avail. The last patient had a sudden cardiac failure while in the respirator.

*Treatment.* Treatment was symptomatic and anxiously expectant during the acute phase. It was found that hot packs relieved the pain of spasm and that pethedine induced comfort and sleep. Initially the limbs were composed in easy extension with a foot board to prevent foot drop. Within the first week of the post-paralytic period physiotherapy was commenced and thereafter all cases have continued under orthopaedic care.

Many of them were actually transferred to an orthopaedic hospital. At first, these transfers were carried out after the patients had been isolated for four weeks—an arbitary figure which was increased to six weeks when two cases developed in the orthopaedic hospital and might have been infected by our transfers. Horstmann (1946) has demonstrated that the virus can be recovered from the faeces for a period of up to two months, although 50 per cent. of cases are clear in less than four weeks from the onset of symptoms.

The mechanical respirator can be a life-saver and was undoubtedly so in several of our cases. The use of this apparatus must be anticipated and it was found very important that the patient be conditioned psychologically in advance if possible. Being stuck into an " iron lung " without warning is a severe shock to most people. The respirator was used as soon as there was evidence of respiratory distress, not waiting until distress had become gross. Discontinuation of the respirator should be as early as is feasible but it should be cautious in order to allow for fatigue in recovering musculature. The respirator is a very specialized instrument and it requires competent supervision by attendants well skilled in its use: constant mechanical care pays good dividends in efficient, trouble-free operation.

In all, seventeen patients made use of the respirator including six of those who died. One small boy required assisted respiration for nearly eight weeks. No patients have needed permanent assistance. At the peak of the epidemic we had four mechanical respirators operating simultaneously.

---

Nobody can write about anterior poliomyelitis without realizing the considerable gaps in our knowledge concerning this disease. These blind spots occur at crucial points in the progress of the disease rendering us helpless to control or cure at the present time. The etiological agent has been long proved and the family tree of the strains of this virus is receiving a great deal of attention: recently Dalldrof *et alii* have extended the family in discovering the Coxsackie virus : Morgan *et alii* have demonstrated the specificity of immunological response to the various strains of the virus. The organism is transmitted and can be recovered from the faeces or, less commonly, the upper respiratory passages (Toomey (1935), Paul (1938), Bodian (1941) ). In this country the clinician gets little help from these developments and, even if he was able to culture the organism, the methods are slow and the results would be retrospective. Active immunity cannot be applied, but if it could be developed by a polyvalent antigen, it might yet control this disease. Physical methods of diagnosis are not accurate and, of course, in this disease the developed illness may always be uninfluenced by treatment, since the crux of the illness is what nerve cells, and how many, have been killed by the infection.

Dead nerve cells will always mean paralysis and, therefore, positive treatment at this stage is impossible. We do not know why the virus prefers to attack motor neurons and we are puzzled why the age incidence is rising in most parts of the world. Little or nothing is known of the factors which decide whether an infection with the virus will result in paralysis or not. We find hope in the very high minor infection rate and are reduced to suggesting that the virus does not really mean to paralyse at all. Yet these unlucky persons who are paralysed increase in numbers every year. Van Riper reports more than 41,500 stricken in the U.S.A. during 1949 and in all probability quite eighty of our small series will carry evidence of morbidity all their lives. While in this hospital, the 1949 cases of poliomyelitis cost us £6,615 and this is only the beginning of the national economic and human loss.

One last problem is the connection between poliomyelitis and the weather. Undoubtedly the summers of 1947 and 1949 were exceptionally fine for Britain and we had plenty of poliomyelitis in each of these summers. This year "as everybody knew would happen"—*Lancet* (1949), the disease fell with the coming of cold weather. Adamson *et alii* however, report severe outbreaks of poliomyelitis among Eskimos in temperatures as low as 81°F, of frost. As a matter of interest, the mean weekly temperatures in Bristol during 1949 are graphed against the weekly admissions of poliomyelitis cases to Ham Green Hospital during the same period.

FIGURE 1.

My thanks are due to H. H. Harding, F. R. Met. Soc., Bristol, for the temperature readings recorded in Fig. I

### REFERENCES

*Lancet*, 1950, **1**, 86.
Casey, A. E., *et al.*—*Amer. J. Dis. Child.*, 1946, **72**, 661.
Howe, H. A.—*Amer. J. Med.*, 1949, **6**, 537.
Horstmann, D. M., *et al.*—*J. Clin. Invest.*, 1946, **25**, 270.
Walldorf, G., *et al.*—*J. Exp. Med.*, 1949, **89**, 567.
Morgan, I. M., *et al.*—*Amer. J. Hyg.*, 1947, **45**, 379.
*Lancet*, 1949, **2**, 1226.
Adamson, J. D., *et al.*—*Canad. Med. Assn. J.*, 1949, **61**, 339.
Van Riper, H. E.—*J. Amer. Med. Assn.*, 1949, Vol. **141**, 1260.

## Figures and Tables

**Figure I. f1:**